# Brain Tumor Networks in Diffuse Glioma

**DOI:** 10.1007/s13311-022-01320-w

**Published:** 2022-11-10

**Authors:** Yvonne Yang, Marc C. Schubert, Thomas Kuner, Wolfgang Wick, Frank Winkler, Varun Venkataramani

**Affiliations:** 1 Neurology Clinic and National Center for Tumor Diseases, University Hospital Heidelberg, INF 400, 69120 Heidelberg, Germany; 2 Clinical Cooperation Unit Neurooncology, German Cancer Research Center (DKFZ), German Cancer Consortium (DKTK), INF 280, 69120 Heidelberg, Germany; 3 Department of Functional Neuroanatomy, Institute for Anatomy and Cell Biology, Heidelberg University, INF 307, 69120 Heidelberg, Germany

**Keywords:** Glioblastoma, Diffuse glioma, Cancer neuroscience, Neuron-glioma synapse, Neuron-tumor networks, Tumor-tumor networks

## Abstract

Diffuse gliomas are primary brain tumors associated with a poor prognosis. Cellular and molecular mechanisms driving the invasive growth patterns and therapeutic resistance are incompletely understood. The emerging field of cancer neuroscience offers a novel approach to study these brain tumors in the context of their intricate interactions with the nervous system employing and combining methodological toolsets from neuroscience and oncology. Increasing evidence has shown how neurodevelopmental and neuronal-like mechanisms are hijacked leading to the discovery of multicellular brain tumor networks. Here, we review how gap junction-coupled tumor-tumor-astrocyte networks, as well as synaptic and paracrine neuron-tumor networks drive glioma progression. Molecular mechanisms of these malignant, homo- and heterotypic networks, and their complex interplay are reviewed. Lastly, potential clinical-translational implications and resulting therapeutic strategies are discussed.

**Supplementary Information:**

The online version contains supplementary material available at 10.1007/s13311-022-01320-w.

## Introduction

Diffuse gliomas are primary brain tumors characterized by their invasive growth [1], colonization of the whole brain [2], and their notorious therapeutic resistance. These brain tumor cells infiltrate into healthy brain parenchyma and reach structures far distant from the main tumor mass, including the brain stem [3], explaining why surgical resection alone cannot completely remove all tumor cells. Standard of care including surgery and radiochemotherapy only results in limited effects on overall survival illustrating the dire need for novel concepts and therapeutic strategies to improve overall outcomes [4, 5].

Increasing evidence emerges showing how neurodevelopmental mechanisms and the nervous system play a pivotal role in brain tumor initiation and progression. Cancer neuroscience provides a novel framework for investigating these intricate relationships between the central nervous system and tumor cells by investigating brain tumors at the interface of neuroscience and oncology [6–9].

Here, we review basic mechanisms of multicellular brain tumor networks [7], their role for glioma biology, their clinical-translational relevance, and potential therapeutic targets.

## Neurite-Like Tumor Microtubes and the Role of Gap Junction-Coupled Brain Tumor Networks

**Fig. 1:**
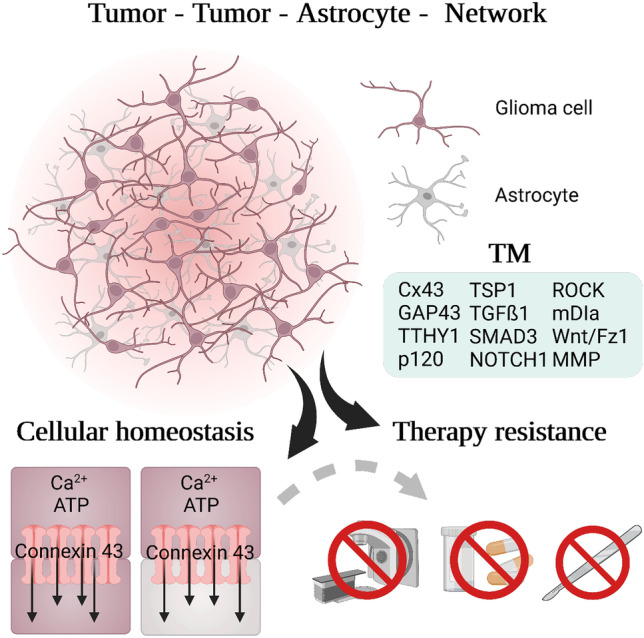
Tumor-tumor networks—molecular driver and biological function. Glioma cells are interconnected via TMs (glioma cells in violet) and integrate themselves into the astrocyte network (astrocytes in grey) to form a therapy resistant glioma cell network, communicating via gap junctions (red). Through gap junctions consisting of connexin 43, they can exchange small molecules (Ca^2+^, ATP) and toxic metabolites to regulate cellular homeostasis

Glioma cells build long membranous protrusions named tumor microtubes (TMs, Fig. 1). These TMs were first visualized with in vivo two-photon microscopy of patient-derived primary glioma cells injected into mice brains [10]. With this method, longitudinal imaging of the same regions revealed that TMs are highly dynamic structures used for scanning the brain [10, 11], invasion [7, 12, 13], and for the formation of a therapy-resistant malignant tumor-tumor cell network [10, 15–17].

Tumor cell networks consist of glioma cells using TMs to interconnect via gap junctions using connexin 43 [10, 18] or adherens junctions [19]. In patient-derived xenograft models, more than 80% of glioma cells had TMs [3]. This coupling of glioma cells enables communication through intracellular calcium waves (ICWs) making them a functional syncytium [10, 13, 19]. Using gap junctions, the tumor cells can exchange small molecules such as calcium ions, ATP, IP3, and microRNA [10]. Additionally, TMs with an average diameter of 1–3 µm can be used to redistribute cell organelles such as mitochondria and nuclei within glioma cells [10, 20]. The cytoskeletal composition in TMs consists of at least both F-actin and microtubules [10].

This tumor network contributes to glioma progression and resistance to all standard of care treatments including surgery, irradiation, and chemotherapy [7, 10, 13, 21, 22]. Functional imaging of glioma cells after irradiation revealed cell death associated with high calcium concentrations of primarily glioma cells which were not connected with each other [10]. In contrast, tumor-connected glioblastoma cells showed lower intracellular concentrations of calcium. This implies that cell death could be potentially prevented by buffering toxic levels of calcium ion concentrations in the gap junction-coupled tumor network. Thus, this gap junction-connected intercellular network can buffer local increases of toxic metabolites by distributing them between large number of cells to avoid cell death. In addition to tumor-tumor connections, glioma cells can additionally integrate themselves into the astrocytic network via gap junctions [23], extending the definition of the malignant network [7, 11] (Fig. 1). The functional role of astrocytes in this network is yet unclear and will need further investigation.

Another way of intercellular membrane tube connection that could contribute to the tumor-tumor-astrocyte network are tunneling nanotubes (TNTs) which were found in adult glioma and many other cancer entities [7, 10, 17, 24–27]. TNTs are predominantly positive for actin and allow transport of cell organelles like mitochondria. With an average diameter of 50–200 nm, they are thinner than tumor microtubes. They can be open-ended at the tip or connected to other cells [27]. In neurodevelopment, calcium communication between astrocytes and developing neurons through tunneling nanotubes (TNT) has been detected [28]. First evidence of TNTs connecting astrocytes and glioma cells was found in in vitro co-cultures [29]. TNTs were formed in reaction to irradiation and TMZ treatment in vitro and patient-derived organoid models [25, 30]. Since TNTs spread O-6-methylguanine-DNA methyltransferase (MGMT) protein and mRNA to other cells [25], they could also serve as anatomical structures contributing to brain tumor networks which will require further investigation.

A similar resistant cellular response to temozolomide was observed in TM-connected tumor cells: TM-rich and interconnected cells resisted the alkylating treatment while tumor-unconnected cells were significantly more sensitive [21].

Dynamic TMs at the invasive front share similarities with axonal growth cones and neurite outgrowth during neurodevelopment [10, 11, 13, 31] which is paralleled by the molecular composition in both anatomical structures at the very tip of the protrusions. The growth-associated protein 43 (GAP43) [10] and tweety-homolog 1 (TTHY1) [13] are important driver genes regulating TM outgrowth and cell invasion into the brain. Additionally, TMs have the striking ability to reach out toward the direction of a surgical lesion to recolonize the injury site [21]. This illustrates the self-repairing mechanisms of this malignant network and its contribution to therapeutic resistance after surgery.

Compared to astrocytoma, 1p19q codeleted oligodendrogliomas are associated with a better prognosis [1]. Genetic analyses revealed that the localization of neurotrophic drivers of GAP43 were found on both 1p19q chromosomal arms [10, 15]. A codeletion of both chromosomal arms led to less TM formation in oligodendrogliomas. In contrast, an overexpression of GAP43 transformed TM-poor oligodendroglioma cells into an interconnected tumor-tumor cell network [10]. Therefore, the occurrence of TMs is correlated with the malignancy of 1p19 non-codeleted gliomas and illustrates the clinical-translational relevance of TM-mediated tumor networks.

Furthermore, P120 catenin was identified as an upstream regulator of GAP43. P120 catenin, also involved in dendritic spine and synapse development [32], was shown to be required for tumor network formation via adherens junctions. The interplay of tumor network connections via gap and adherens junctions is yet unclear and will allow further insight into basic properties of these networks.

In addition, TM formation can be driven by other molecular drivers, some of which are relevant for neurite development in the central nervous system [33–35]. For example, transforming growth factor ß (TGF-ß) increases neurite outgrowth in the developing brain and similarly led to increased TM formation with mothers against decapentaplegic homolog 3 (SMAD3) and thrombospondin-1 (TSP-1) as downstream mediators [36]. Similarly, inhibition of mammalian diaphanous-related (mDIA) formin reduced TM formation [37]. It will be important to understand the role of these molecular drivers to therapeutic resistance in future studies.

## Synaptic Neuron-Tumor Networks Promote Glioma Proliferation and Invasion

**Fig. 2:**
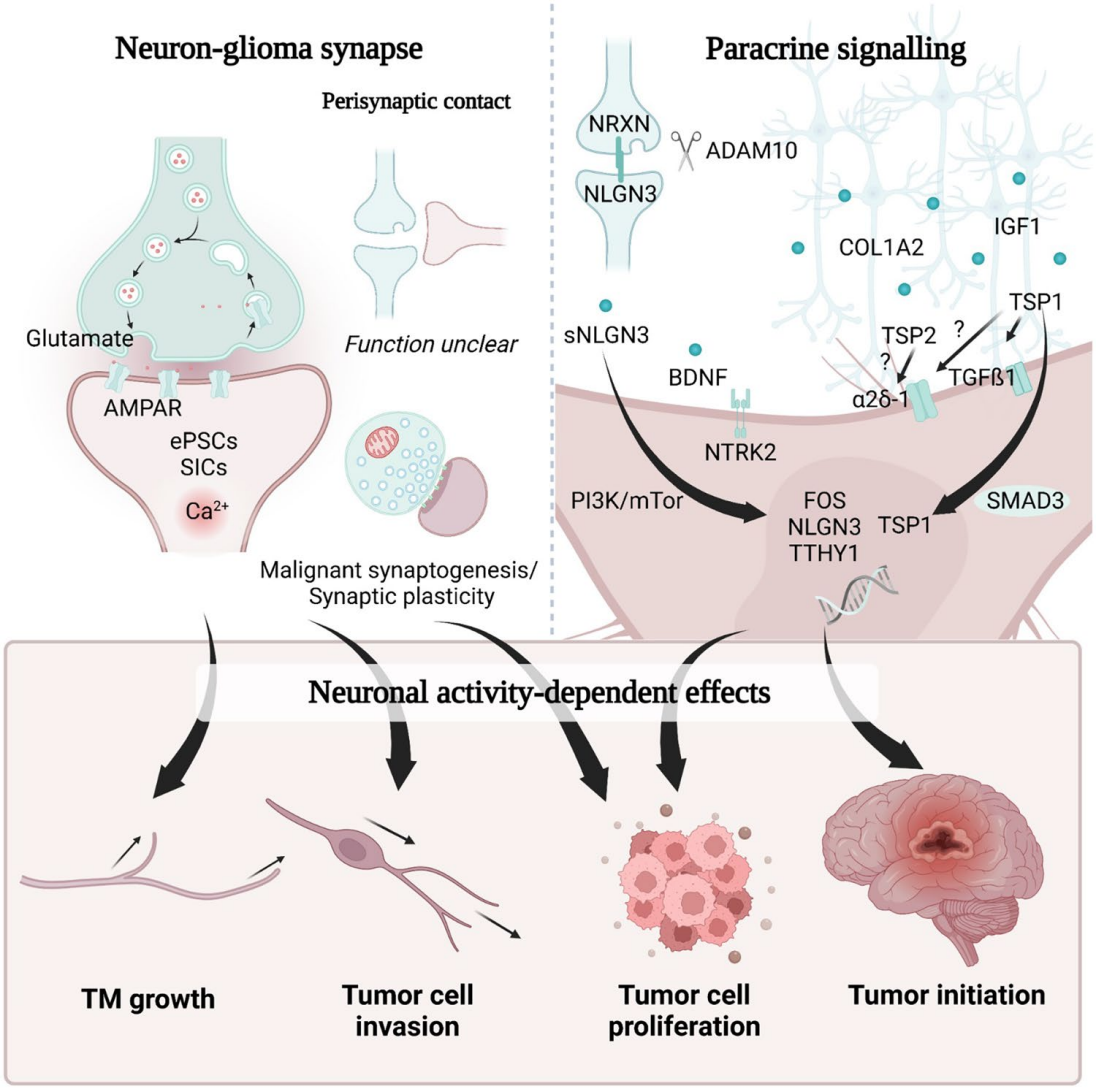
Neuron-glioma networks molecular mechanisms and biological functions. Neuron-tumor communication is based on synaptic and paracrine pathways. Glutamatergic neuron-glioma synapse communication is mediated via AMPARs. *EPSC* excitatory postsynaptic currents, *SIC* slow inward currents. Perisynaptic glioma cell mediates synaptic transmission of physiological synapses, but their function is yet unclear. Paracrine signaling via NLGN-3, BDNF, IGF-1, COL1A2, and TSP-1 mediates paracrine neuron-to-tumor signaling. Neuronal input drives tumor cell invasion, TM growth, new formations of synapses, proliferation, progression, and tumor initiation

With the growing understanding of tumor integration into the malignant network, new direct and indirect pathways of communication with neurons have been discovered (Fig. 2). Testing the effects of neuronal activity on glioma cell proliferation in patient-derived xenograft models revealed a significant increase of the tumor cell proliferation index that could be observed within 24 h [44].

Morphological analysis of glioma cells and neurons revealed that TMs are the predominant location of neuronal interaction illustrating another important function of TMs [11]. A closer look with ultrastructural analyses revealed a heterogenous synaptic integration of glioma cells into the neuronal network [12, 14].

Apart from the gap junction-coupled tumor network, *bona fide* glutamatergic synapses formed between neurons and glioma cells were found in tumor subpopulations of adult isocitrate dehydrogenase wildtype (IDH-wt) glioblastomas, adult isocitrate dehydrogenase mutant (IDH-mut) astrocytoma [12, 14], and pediatric histone-H3 mutant (H3K27M) diffuse midline gliomas (DMG) [14]. Then, 10–20% of electron microscopic sections of tumor cells showed neuron-glioma synapses [12]. As a single glioma cell cannot be completely captured by a single electron microscopic section, the exact numbers of synapses per glioma cell have not yet been determined. Neuron-glioma synapses (NGS) have not yet been detected in preclinical brain tumor models that are correlated with a better prognosis than diffuse gliomas such as meningiomas or oligodendrogliomas [12]. Presynaptic neuronal glutamate release leads to excitatory postsynaptic currents (eEPSCs) via α-amino-3-hydroxy-5-methyl-4-isoxazolepropionic acid receptor (AMPAR) and slow inward currents (SICs) in the postsynaptic glioma cell [7, 12, 14, 38]. These electrical currents could induce calcium transients in glioma cells [7, 12, 14, 38] that in turn promoted TM dynamics, tumor cell invasion, and tumor cell proliferation [7, 10–14, 18]. In pediatric diffuse midline gliomas, neuronal activity-induced SICs were mediated by potassium channels [14]. In adult gliomas, an interplay of potassium currents [12, 14], AMPAR, and glutamate transporters [12, 38] might be relevant for SICs which will need further characterization. Unraveling molecular and downstream mechanisms as well as understanding the functional role of SICs for glioma biology will be important to characterize another layer of neuron-glioma communication.

Apart from direct synaptic contacts, perisynaptic glioma cells contacting physiological synapses were also found in glioblastoma [12], but their function remains unclear. Malignant perisynaptic contacts play an important role in brain metastases [39], since these are associated with *N*-methyl-d-aspartate receptor (NMDAR)-dependent brain metastatic growth [39]. However, in glioma, there is no clear evidence for NMDAR signaling [12, 14]. Therefore, the function of perisynaptic contacts needs to be explored further in the future.

## Paracrine Neuron-Glioma Communication Drives Tumor Initiation and Glioma Cell Proliferation

Apart from direct synaptic and perisynaptic communication, paracrine signaling is another important layer of neuron-glioma communication. Paracrine factors are released in the tumor microenvironment, influencing tumor progression by promoting high-grade glioma (HGG) growth and proliferation (Fig. 2) [14, 40–47].

The synaptic protein neuroligin 3 (NLGN-3) is secreted by neurons and oligodendrocyte precursor cells (OPCs) in a neuronal activity-dependent manner [48]. After cleavage by the metalloproteinase ADAM10, soluble NLGN-3 (sNLGN-3) binds on glioma cells leading to PI3K-mTOR signaling activation [43–45]. This in turn could initiate tumor formation in a genetic predisposition syndrome called neurofibromatosis type 1 (NF1). Furthermore, it could promote tumor growth in both NF1-associated gliomas and other gliomas [41–43, 46, 47]. On an expression level, NLGN-3-associated PI3K-mTOR signaling in glioma cells led to an upregulation of synapse-associated genes [45]. In addition, high levels of NLGN3 expression in human HGG correlated negatively with patient overall survival [44]. Simultaneously, it was shown that glioma cells can also secrete NLGN-3 into tumor microenvironment, which was reciprocally regulated by neuroligin-exposure from the tumor microenvironment [45]. Interestingly, another downstream mechanism of NLGN-3 is the upregulation of TTYH1 expression, a driver of TM formation [13] suggesting a potential role of NLGN-3 for TM genesis and growth.

In addition to NF-1 associated optic nerve gliomas in which visual stimulation could drive tumor initiation, olfactory sensory experience could also promote gliomagenesis. This was driven by the neuronal activity-dependent paracrine secretion of insulin-like growth factor 1 (IGF-1) in mitral and tufted cells. Additionally, IGF-1 secretion promotes tumor cell proliferation and tumor growth [49]. Synapse and neurotransmitter related genes are upregulated in AC-like and OPC-like tumor cells of a particular tumor model [49, 50]. However, the suggested independence of both IGF-1 and synaptic pathways was based on in vitro co-culture experiments [49]. It will be interesting to understand how IGF-1 secretion might modulate neuron-glioma synapses in other model systems with a neural microenvironment.

Brain-derived neurotrophic factor (BDNF) is a neurotrophine known for its stimulating effect on neuronal growth and synapse formation [51]. Analogously, BDNF promotes malignant synaptic plasticity and increases calcium transient intensity under glutamate stimulation in HGG [41]. Additionally, BDNF regulates trafficking of AMPAR to the postsynaptic membrane in glioma cells and promotes glioma progression through neurotrophic tyrosine kinase receptor 2 (NTRK2) in a neuronal activity-dependent manner. Both knockdown and pharmacological targeting of NTRK2 showed prolonged median survival in patient-derived xenograft mouse models [41]. Interestingly, NTRK2 expression correlated with GJA1 and TTYH1 expression [41].

The glycoprotein thrombospondin-1 (TSP-1/THBS1) binds to α2δ-1 and regulates neural progenitor cell (NPC) differentiation and proliferation [52]. It belongs to the astrocyte-derived neurogenic factors regulating synapse formation and spinogenesis [52]. In the malignant context of primary brain tumors, TSP-1 expression is activated by TGF-ß1 which leads to formation of TMs. Also, via TGFß1-SMAD signaling, THBS1 expression is upregulated [53], causing a positive feedback loop (Fig. 2). A knockdown of TSP-1 showed reduced TM formation and inhibited glioma cell invasion [36].

Human tumor tissue areas with high neuronal functional connectivity, based on electrocorticography (ECOG) and MEG, showed an upregulation of TSP-1 expression in RNA-seq data and on a protein level compared to glioma regions with lower neuronal functional connectivity. Additionally, biopsies from regions with high functional connectivity were increased for the postsynaptic protein PSD95 compared to the low functional connectivity regions. Interestingly, patients with tumor-infiltrated brain regions with a high neuronal functional connectivity had a worse survival further indicating an important role for neuron-tumor networks influencing glioblastoma biology.

Lastly, glioma cells could even utilize Wingless (Wg)-related integration site (WNT) pathway from neurons that promoted glioblastoma progression in a *Drosophila* model. This simultaneously led to neurodegenerative effects due to depletion of Wg [54], highlighting the importance of investigating bidirectional effects of neuron-tumor networks.

To summarize, several molecular mechanisms of paracrine and synaptic neuron-glioma interactions driving glioma progression have been identified. How they interdepend and interact with gap-junction mediated heterogeneous tumor networks are still questions to be answered that will deepen our understanding of these complex interactions.

## Interplay of Glioma Cell State, Connectivity, and Biological Function

As described above, multicellular tumor networks have been described as a hallmark of malignant brain cancers. However, it was not yet clear how synaptic input, tumor-tumor cell networks, cellular heterogeneity including neuronal-like transcriptomic cells states, and biological function are exactly interrelated.

Based on their cell morphology, glioma cells can be classified concerning their integration into the gap junction-mediated TM network (Fig. 1) [10–12, 22, 55, 56]. Molecularly, glioblastomas have been categorized with several classifications using single-cell RNA-sequencing. Cellular states based on gene expression patterns [50] or pathway activity [57] have been proposed. A prominent gene expression classification categorizes into neural progenitor-like (NPC-like), oligodendrocyte-progenitor-like (OPC-like), astrocyte-like (AC-like), and mesenchymal like (MES-like) states [50]. Complementary, cells can be grouped based on their pathway activity into glycolytic/plurimetabolic (GPM), mitochondrial (MTC), neuronal (NEU), and proliferative/progenitor (PPR) cell states [57]. Additionally, glioblastoma cells can be integrated into a gradient from expressing neurodevelopmental genes (Developmental) to expressing genes for inflammatory wound response (Injury) [58].

**Fig. 3:**
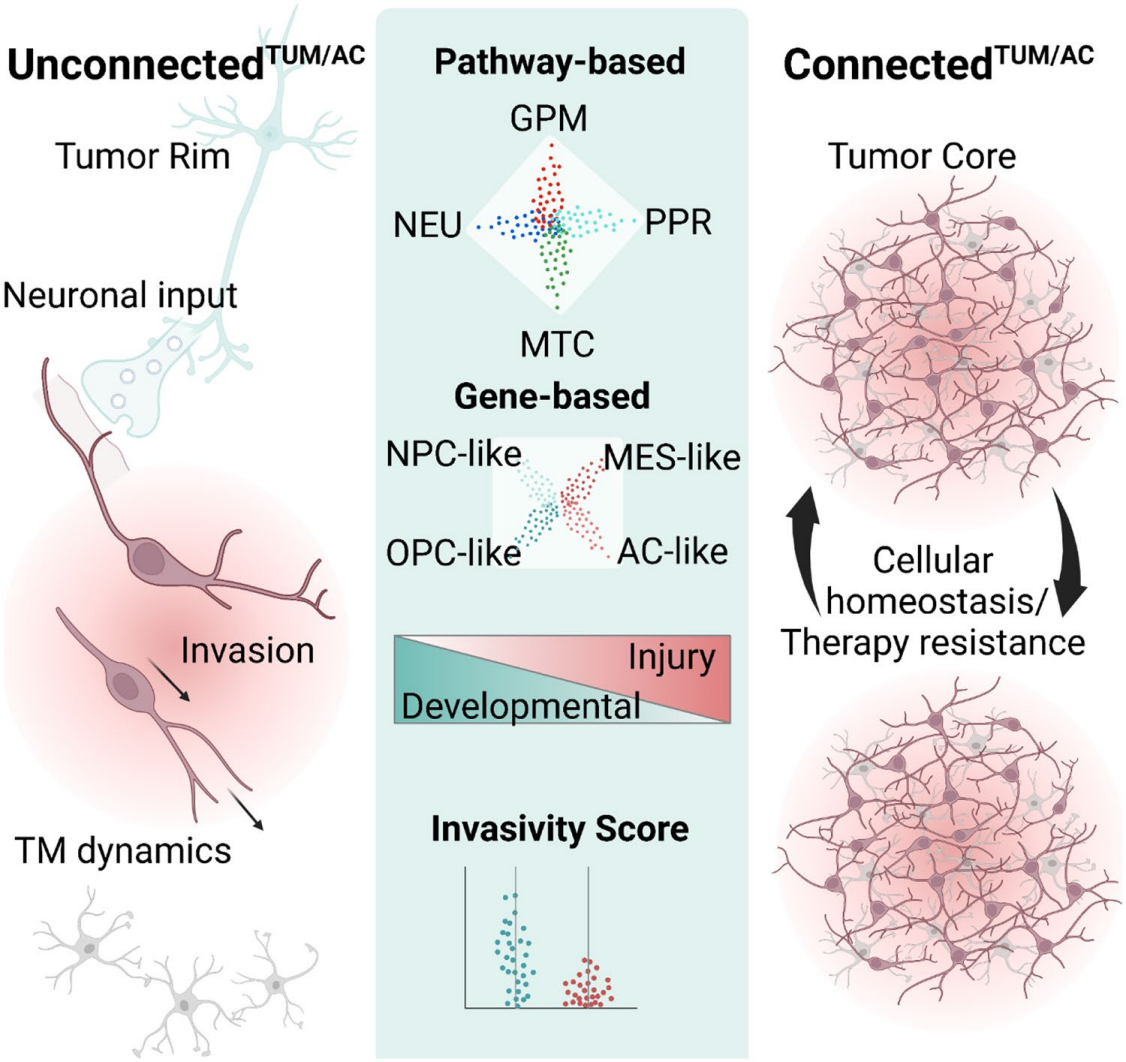
Glioma cells hijack neuronal-like mechanisms for cell invasion. By integrating transcriptomic signatures, cellular behavior, localization, and cell connectivity, two poles of cell states can be identified. Molecular signature of the neuronal-like subtype shows distinct characteristics in transcriptomic based classifications: NEU/NPC/OPC/Neurodevelopmental/high invasivity score. These cells are primarily unconnected^TUM/AC^, highly invasive and to be found at the tumor rim. Network-integrated connected^TUM/AC^ cells in the tumor core, in contrast, form a tumor-tumor-astrocyte network and show transcriptomic signatures of AC-like/MES-like/Injury/low Invasity score

An integration of transcriptomic [50, 57, 58], tumor cell localization within glioblastomas [59], connectivity-based characteristics, and cellular behavior [11] displayed a transcriptomic gradient along two opposing cell states with different biological functions. The subgroup of tumor cells not connected to other tumor cells or astrocytes (TUM/AC) predominantly found in the tumor periphery (Fig. 3) showed an enrichment for OPC/NPC-like cell state expression [50], NEU pathway-based signaling [57], and neurodevelopmental signatures [58]. In contrast, cellular states of highly connected^TUM/AC^ glioma cells which were mostly found in the tumor core [59], often shared AC/MES-like states [50] and injury response signatures [11, 18, 58]. The connected^TUM/AC^ tumor cells form the malignant tumor network and communicated with astrocytes using gap junctions, while unconnected^TUM/AC^ cells are the driver of glioma cell invasion [11]. These new findings are in disagreement with previously suggested mechanisms of glioma cell invasion such as collective migration and the mesenchymal cell state being the driver of invasion [19, 60–62]. Instead, primarily unconnected^TUM/AC^ glioma cells hijack neuronal-like mechanisms (Fig. 3) to colonize the brain. Especially the dynamic movements of their TMs, their branching behavior, as well three migration phenotypes called locomotion, branching migration, and translocation are comparable to neuronal migration during development [63–68]. Correlative ultrastructural analyses revealed that neuron-glioma synapses can be found on connected^TUM/AC^ as well as on unconnected^TUM/AC^ tumor cells [11]. Therefore, both morphological subgroups could receive neuronal input. In the unconnected^TUM/AC^ subgroup, neuronal activity increased glioma cell invasion and TM dynamics. Therefore, unconnected^TUM/AC^ tumor cells do not only show transcriptomic and morphological neuronal signatures, but their invasion is also driven by neuronal activity. The potentially distinct biological effects of synaptic input on unconnected^TUM/AC^ and connected^TUM/AC^ subgroup should be investigated in the future.

A critical question for understanding glioma heterogeneity is how cell states change over time. To investigate tumor evolution on a molecular level, pseudotime analysis [69] can be performed on transcriptomic data. In parallel, two-photon microscopy of tumor-bearing mice with chronic cranial windows allows to analyze changes of the same brain microregion over weeks. The integration of both aspects revealed that unconnected^TUM/AC^ cells and tumor cells with the NEU state are predominantly found in earlier stages and as the tumor progresses, glioma cells become more connected^TUM/AC^ with a shift to AC-like/MES-like and GPM/MTC states [11]. It will be important to investigate aspects of tumor evolution on a single-cell level, both with integrated imaging and molecular analyses, to completely reveal the spectrum of potential cell states and mechanisms of exact cell state evolution. Although this will necessitate further technological developments, it offers the promise of characterizing the dynamic pathobiology of diffuse glioma and potential novel clinical-translational concepts.

In accordance with data from xenograft and resected human material from glioblastoma patients, a recent study showed that cancer cells in recurrent glioblastomas enriched for neuronal signaling were associated with more infiltrative growth [70]. These data clearly support the correlation of neuronal tumor phenotypes that drive glioblastoma invasion.

In summary, neuron-glioma synapses mediate proliferation, invasion, and TM generation and dynamics. While we understand that invasion and TM generation as well as dynamics are mediated by synaptic input on unconnected^TUM/AC^ brain tumor cells, the role of synaptic input to connected ^TUM/AC^ cells building up the brain tumor network is yet unclear. Further investigation into the exact trajectories of tumor evolution, malignant synaptic plasticity, and their biological function including therapeutic resistance are important next steps.

## A Potential Vicious Circle of Neuronal Hyperexcitability and Glioma Progression

**Fig. 4:**
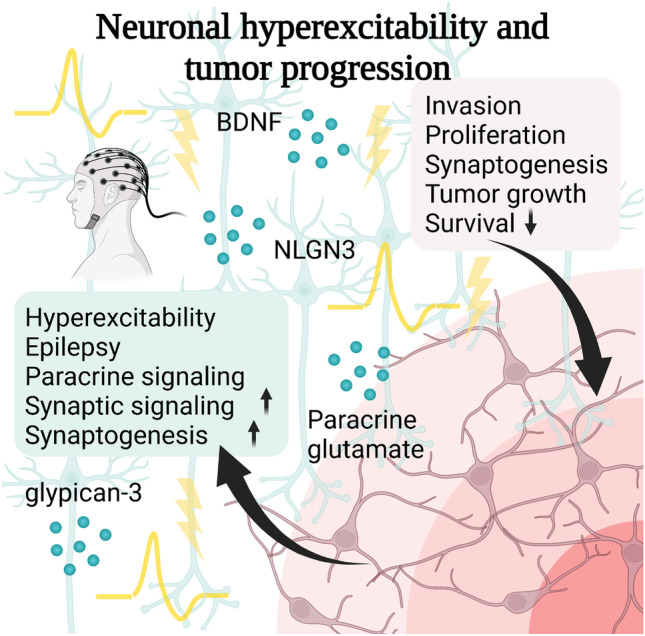
Neuronal hyperexcitability and remodeling. Bidirectional communication between neurons (blue) and glioma cells (violet) constructs a vicious circle leading to neuronal hyperexcitability (yellow). Reciprocal influence between glioma cells and neurons can lead to remodeling of both glioma cell and neuronal network

Neurons and glioma cells can reciprocally alter each other’s functions illustrating that neuron-glioma communication is bidirectional. As gliomas induce neuronal hyperexcitability, epilepsy is a common comorbidity in patients (Fig. 4) [7, 38, 71].

The interplay between both neuronal hyperexcitability and glioma progression has been demonstrated for certain PI3KCA (phosphatidylinositol-4,5-bisphosphate 3-kinase catalytic subunit alpha) variant tumors. Several variants of PI3CKA in glioblastoma cells modulate the expression of synaptic profiles leading to an increase in hyperexcitability and gliomagenesis mediated by glypican-3 secretion [72]. Other neuronal activity-dependent paracrine signaling pathways involving NLGN-3, BDNF, 78 kDa glucose-regulated protein (GRP78) [14, 44, 45], IGF-1 [49], and COL1A2 [46] could be potentially upregulated by neuronal hyperexcitability. Glioma-derived thrombospondine 2 (TSP-2) leads to an increase in excitatory synapse formation and elevates epileptiform discharges in the peritumoral regions [73]. Also, paracrine secretion of glutamate by glioma cells can contribute to neuronal hyperexcitability through the cysteine-glutamate transporter system [74]. As this transporter system can be pharmacologically targeted by sulfasalazine, neuronal hyperexcitability and tumor growth could be potentially reduced [75].

Preliminary studies have also shown that glioma cells communicate with the brain by shedding extracellular vesicles (EV) [76] and that EV-mediated communication seems to increase synaptic activity in neurons. Reciprocally, the inhibition of EV release reduces glioma growth suggesting EVs to be a potential therapeutic target.

Seemingly contrary to a potential association between neuronal activity and tumor progression, oligodendroglioma patients with seizures showed a more favorable outcome in survival than those without [77]. However, the difficulty to interpret results regarding the association between seizures and survival in brain tumors should be taken in consideration. First, low-grade gliomas grow more slowly and can therefore disturb neuronal circuits over a longer time [38]. Due to different time points of diagnosis, the possibility for a significant lead time bias should not be overlooked [78]. Additionally, the investigation of clinically observable seizures as only readout fails to detect the relevance of potential subclinical neuronal hyperexcitability which might play an important role in tumor pathophysiology.

Recently, clinical data suggested how neuronal hyperexcitability and glioma progression might be interrelated. The authors showed that the occurrence of status epilepticus was correlated with poorer survival in glioblastoma patients in a retrospective study [78] indicating that indeed neuronal hyperexcitability is a prognostic factor in these incurable brain tumors. Additionally, it will be important to monitor subclinical neuronal hyperexcitability as this might still affect glioma growth which will require further examination via longitudinal monitoring via EEG and MEG [38]. Additionally, it has been recently reported that gliomas occur more often in brain regions with higher activity [79].

Taken together, increasing evidence shows a bidirectional interplay between neurons and glioma cells which could lead to a positive feedback loop and subsequent vicious circle [38]. The reciprocal effects between neurons and glioma cells will need further preclinical and clinical investigation to reveal the exact mechanisms of action.

## Targeted Therapy of Brain Tumor Networks

Several concepts for pharmacological disconnection from neuron-tumor and tumor-tumor networks have been demonstrated in preclinical models and early-phase clinical studies. Understanding their relevance in clinical translation should be the next step. In particular, the molecular, cellular, and spatial heterogeneity of these tumors including their plasticity and evolution over time need to be taken in account.

As therapeutic targets aim at disconnecting brain tumor networks, it is important to understand therapeutic windows that halt brain tumor progression while preserving the integrity of the central nervous system. This will necessitate the dedicated study of therapeutic effects on the central nervous system, an important research area of cancer neuroscience [6, 35, 80].

One therapeutic approach could be the inhibition of neuronal activity with antiepileptic therapy to disrupt synaptic and paracrine communication between neurons and glioma cells. In multiple studies, no prolongation of overall survival was observed in patients receiving anticonvulsants [81, 82]. However, only pharmacological agents affecting presynaptic mechanisms were investigated and the effect on potential subclinical hyperexcitability that still could contribute to tumor progression were not considered [12, 77].

Neuron-glioma glutamatergic synaptic transmission can be blocked by AMPAR inhibitors such as talampanel or perampanel. These non-competitive AMPAR antagonists are well-tolerated antiepileptic drugs (AED).

Before its discontinuation due to its short half-life, talampanel was tested in its antitumoral effects as an add-on drug for glioblastoma in two clinical trials. A smaller trial phase II trial investigated patients with recurrent glioblastomas regarding their median survival. Talampanel was given in parallel to standard of care adjuvant therapy and failed to show its effect in prolonging survival [83]. In contrary, talampanel was evaluated in newly diagnosed glioblastoma patients in a multi-centric phase II trial. Both patients with methylated and unmethylated *MGMT*-status showed prolonged survival compared to the control group only receiving radiochemotherapy. Surprisingly, the talampanel group showed prolonged survival despite the higher percentage of patients with an unmethylated MGMT status [84, 85]. The fact that only historical controls were used impedes the interpretation of both clinical trials. Due to the unfavorable pharmacokinetics of talampanel, other AMPAR inhibitors might be more appropriate to reproduce the results and to investigate the effects of AMPAR signaling.

As for the FDA-approved noncompetitive AMPAR inhibitor perampanel, antitumoral cellular effects were described in several preclinical studies in treatment of adult and pediatric high-grade gliomas [12, 14, 86, 87]. Effectively, perampanel inhibited proliferation [14], cell invasion at the infiltrative tumor edge [11, 12], as well as TM formation and dynamics [11].

In clinical trials, perampanel showed favorable pharmacokinetics in comparison to talampanel and its effectiveness as an antiepileptic drug in glioblastoma patients [88]. Currently, the effect of perampanel on peritumoral hyperexcitability and its reduction on seizure frequency are studied (NCT04497142 and NCT04650204) [89]. In addition, it will be important to study tumor-specific and microenvironmental effects of perampanel in a randomized, multi-centered study as planned for the window-of-opportunity trial PERSURGE [7].

As pan-AMPAR inhibitors might also limit the therapeutic window, more specific inhibitors of neuron-glioma synapse need to be further explored. It has been shown that AMPAR in gliomas are at least partially calcium-permeable [12, 14]. Specific targeting of calcium permeable AMPARs [90] could be achieved by using e.g. IEM1460 which was investigated for its antiepileptic effect but not examined in preclinical and clinical treatment of gliomas yet [91].

Furthermore, neuronal paracrine signaling can also be targeted through inhibition of NLGN-3 shedding. ADAM10, a metalloprotease which increases NLGN-3 release [43–45], can be inhibited with the agents GI254023X and INCB7839. In a DIPG xenograft mouse model, the ADAM10 inhibitors decreased tumor cell proliferation and subsequent tumor growth. Clinical application of INCB7839 is currently investigated in a phase I study in treatment of recurrent or progressive pediatric HGGs (NCT04295759).

Another way to inhibit neuronal paracrine signaling is to target the effects of BDNF in the tumor microenvironment. Entrectinib is a pan-Trk inhibitor which also interacts with ROS1 and ALK pathways [92]. TrkB, encoded by *NTRK2*, is the specific receptor of BDNF and can be inhibited pharmacologically with this drug. In a preclinical xenograft DIPG model, malignant trafficking of AMPARs on the postsynaptic membrane and tumor proliferation was inhibited which led to increased survival. Another drug effect was the increase of GLU4R phosphorylation, which potentially could lead to upregulated formation of neuron-glioma synapses [41]. In vitro studies also indicate an antiproliferative effect of entrectinib on low grade optic glioma in the NF1 genetic model [47]. Apart from targeting the neuronal BDNF pathway, entrectinib gains importance in therapy and diagnostics of CNS tumors: The 2021 WHO classification identifies a new class of infant-type hemispheric glioma presenting with receptor tyrosine kinase fusions with *ALK*, *ROS1*, *NTRK1/2/3*, or *MET* [1]. The influence of *NTRK* fusions or alterations as a prerequisite for effective treatment of entrectinib needs to be further investigated as these pathways might also play a role in brain tumors without these fusions potentially targeting neuron-tumor networks. Furthermore, the use of the selective pan-Trk inhibitor larotrectinib could be considered with respect to these pathways [93, 94].

Another clinically relevant target in the context of cancer neuroscience could be thrombospondins. This class of molecules has many different receptors, but the most promising target is α2δ-1-R [52, 95], subunit of T-type voltage-sensitive calcium channel (VSCC) since it mediates synaptogenesis and spinogenesis and could be targeted with FDA-approved drugs such as gabapentin and pregabalin [73]. Apart from α2δ-1-R targeting, TSP1 seems to be involved in TGF-ß1/SMAD3 signaling which in turn increases TSP1 expression. This could potentially lead to increased TM formation [7, 10, 36]. Brain-penetrant and tolerable agents disrupting TSP/TGF-ß1/SMAD3 are yet to be identified. An inhibition of the TSP1-mediated pathway could disrupt both tumor-tumor and the neuron-tumor communication by decreasing TM formation, malignant calcium communication [36], functional network communication [40], and potentially inhibiting malignant synaptogenesis [52]. The blood–brain barrier permeable small molecule inhibitor of IGF-1-R, picropodophyllin (PPP) [96], shows promising antitumoral effects in treatment of gliomas [97] and could be used for another way of disrupting paracrine neuron-tumor signaling. The pharmacological inhibition of IGF-1-R led to induction of apoptosis, inhibition of growth and subsequent tumor size reduction [49, 97]. A single-center non-randomized phase I trial (*n* = 9) tested PPP on patients with recurrent astrocytomas, mostly glioblastomas (NCT01721577) [98]. PPP was well-tolerated except for reported dose-limiting effects such as neutropenia and thrombocytopenia in some cases. Since a new oral formulation of AXL1717 (PPP) is on the way, this could be considered for clinical investigation of its anti-tumoral effects [98].

Lastly, disconnection of tumor-tumor networks connected via gap junctions has been shown to reduce therapeutic resistance of gliomas [7, 10, 15, 21, 38, 99, 100]. Meclofenamate (MFA), for instance, is a gap junction inhibitor and primarily known as a U.S. Food and Drug Administration (FDA)-approved nonsteroidal anti-inflammatory drug (NSAID) in the treatment of rheumatic arthritis. Repurposing this drug for the treatment of glioma, MFA sufficiently disrupts TM networks by decoupling gap junctions and in turn has been shown to prolong survival in animal studies. This drug is currently being investigated clinically in the MecMeth trial as an add-on drug to standard radio-and chemotherapy [101].

To overcome therapeutic resistance against TMZ, INI-0602, a newly developed blood–brain-barrier permeable gap junction inhibitor, sensitized glioma cells to chemotherapy [102]. Further development of TM network-disrupting drugs [103] could be an interesting pharmacological strategy to overcome therapeutic resistance in recurrent gliomas.

## Discussion

In summary, we have reviewed evidence of brain tumor networks contributing to various functions of cancer biology. Neurodevelopmental and neuronal-like mechanisms are hijacked by brain tumors that show an intricate interplay with the central nervous system and could be involved in a vicious cycle of neuronal hyperexcitability and glioma progression.

However, this also offers novel therapeutic opportunities. Therefore, pharmacological targets of tackling brain tumor networks are discussed with their potential caveats.

Taken together, the strategy of disconnecting these homo- and heterotypic networks can lead to a paradigm shift in developing therapeutic strategies for incurable brain tumors.

## Supplementary Information

Below is the link to the electronic supplementary material.

Supplementary file1 (PDF 421 kb)

Supplementary file2 (PDF 384 kb)

Supplementary file3 (PDF 374 kb)

Supplementary file4 (PDF 393 kb)

Supplementary file5 (PDF 383 kb)

Supplementary file6 (PDF 508 kb)
